# Current News

**Published:** 1896-11

**Authors:** 


### Current News.

### PHILADELPHIA COUNTY DENTAL SOCIETY.

At a meeting of the Philadelphia County Dental Society, held October 17, 1896, the following resolutions were adopted:

WHEREAS, The Pennsylvania State Dental Society is a delegated body, made up from representatives of local societies in the State; and,

WHEREAS, The Philadelphia County Dental Society is one of these representatives, and it therefore becomes the Society's privilege and duty to take part in all that concerns the welfare of the Pennsylvania State Dental Society; and,

WHEREAS, At the meeting held at Bellefonte, Pa., July 7, 8, and 9, 1896, a crime was committed that, if allowed to pass unrepudiated, will, in the future, place a stain upon the name of dentistry in Pennsylvania; therefore, be it

*Resolved*, That this Society strongly denounces such proceedings, and urges that the guilty be punished.

